# Selection of Urbanized Areas by Magpie *Pica pica* in a Medium Size City in Poland

**DOI:** 10.3390/ani11061738

**Published:** 2021-06-10

**Authors:** Olaf Ciebiera, Paweł Czechowski, Federico Morelli, Robert Piekarski, Marcin Bocheński, Justyna Chachulska-Serweta, Leszek Jerzak

**Affiliations:** 1 Institute of Biological Sciences, University of Zielona Góra, Prof. Z. Szafrana Str. 1, 65-516 Zielona Góra, Poland; p.czechowski@wnb.uz.zgora.pl (P.C.); fmorellius@gmail.com (F.M.); m.bochenski@wnb.uz.zgora.pl (M.B.); chachulska.j@gmail.com (J.C.-S.); l.jerzak@wnb.uz.zgora.pl (L.J.); 2 Faculty of Environmental Sciences, Czech University of Life Sciences Prague, Kamýcká 129, CZ-165 00 Praha-Suchdol, Czech Republic; 3 Association Graduates of the AWF Gorzów Wielkopolski, Estkowskiego Str. 13, 66-400 Gorzów Wielkopolski, Poland; robpieka@wp.pl

**Keywords:** magpie, *Pica pica*, urban population, habitat selectivity

## Abstract

**Simple Summary:**

The aim of this study was to estimate the Magpie population and to give a detailed characterization of nest site selection in a medium size city in Poland (Gorzów Wlkp.) in the XXI century. We also focused on the analysis of nest site selection along an urban gradient. The average density of Magpies was 5.5 pairs/km^2^ (min = 0, max = 22 nests/square). The Magpie density in those study squares situated completely within the city boundaries of Gorzów Wlkp. was 6.9 p/km^2^, in the non-urbanized habitat type—3.7 p/km^2^, and in the urbanized habitat type—13.5 p/km^2^. The increasing proportion of the number of nests along the urbanization gradient shows that in Gorzów Wlkp., the Magpie population has undergone the synurbanization process and is currently in the “spread” phase. The study shows that Magpies can adapt to changing urbanization factors, and changes in the choice of conifers help the species to adapt to highly anthropogenic habitats.

**Abstract:**

The Magpie *Pica pica* occurs all over open agricultural areas in Poland, especially near human settlements (particularly in western Poland). The aim of this study was to estimate the size of the local Magpie population and characterize, in detail, nest site selection in a medium size city Górzów Wlkp. in the XXI century. For this study, the whole city was divided into a total of 114 squares of 1 × 1 km. Data were collected in spring 2014. A total of 474 Magpie pairs were recorded. The average density was 5.5 pairs/km^2^ (min = 0, max = 22 nests/square), in the non-urbanized habitat type—3.7 p/km^2^, and in the urbanized habitat type—13.5 p/km^2^. Magpie nests were found most often on Spruces *Picea* sp. and Poplars *Populus* sp. The mean height of the nest site was 11.5 m, while the mean height of trees used for nesting was 13.4 m. The type of tree arrangement most frequently used for nesting was tree rows (26.3%), followed by single trees (24.6%) and clusters of 4–10 trees (20.1%). The results for the Magpie’s environmental preferences do not differ from the general patterns described earlier. The study shows that magpies can adapt to changing urbanization factors, and changes in the choice of conifers help the species to adapt to highly anthropogenic habitats.

## 1. Introduction

The Magpie *Pica pica* occurs all over open agricultural areas in Poland, especially near human settlements (particularly in western Poland). The entire breeding population is estimated to be 360–410 thousand pairs [1]. In the beginning of the XXI century, the population increased slightly [2]. The density in urban areas even reaches several dozen pairs per km^2^ in big cities in Poland, e.g., Zielona Góra, Wrocław, Warszawa, Kraków, Gdańsk, and Poznań [2]. In Eastern Poland, the densities in cities are slightly lower, as shown for Olsztyn, Siedlce, Biała Podlaska, and Białystok [3,4,5]. The Magpie was rarely observed in cities until the 1st half of the XX century [6]. In the 2nd half of the XX century, the numbers in urban areas strongly increased in European and Asian cities. Since this period, the Magpie has been of high interest for ornithologists because this Corvid species can have an impact on songbird (Passeriformes) numbers and has been considered an important predator of ground-nesting birds [7,8]. Hospitable management of green areas can avoid a human-induced regulation of particular bird populations in urban areas [9]. Finding a suitable nest site is a major factor for tree-nesting birds to reproduce successfully, especially in urban environments where suitable nest sites are usually reduced [9,10]. Even though deciduous trees are still favoured over conifers for nest building, there is a trend for the Magpie to build nests in evergreen tree species, which may contribute to the significant expansion of the Magpie to city centres. Papers from recent years indicate that the share of conifers as a nesting site is increasing, especially in the leafless period in the early stage of spring [3,5,11]. This is one reason to study the habitats and nest site selection of Magpies and others bird species in urban areas. Studies in recent years have shown an increasing number of Hooded crows, *Corvus cornix*, in urban areas and its interaction with Magpie populations [12,13,14] and a still increasing proportion of Magpies nests built in conifers [5,11].

The aim of the present study was to estimate the Magpie population and characterize in detail nest site selection in a medium size city in Poland in the XXI century. We also focused on the analysis of nest site selection along an urban gradient due to lack of sufficient knowledge in the field of selectivity of the Magpie’s nesting places. Publications from recent years indicate that the magpie is concentrated in the centres of large cities and leave the semi-open areas of the suburbs for highly urbanized areas [11,15].

## 2. Materials and Methods

### 2.1. Field Study

Gorzów Wlkp. (52°43′51″ N, 15°14′18″ E) is a medium size city in north-western Poland with 125,000 inhabitants. It is located in the Warta valley and covers an area of 85.72 km^2^ in the Lubuskie State. The surrounding landscape of Gorzów Wlkp. is dominated by a mosaic of agricultural and forested areas. The Warta River flows through the city. The climate of Gorzów Wlkp. is temperate with an annual mean temperature of 9.1 °C and a precipitation of 544 mm.

**Figure 1:**
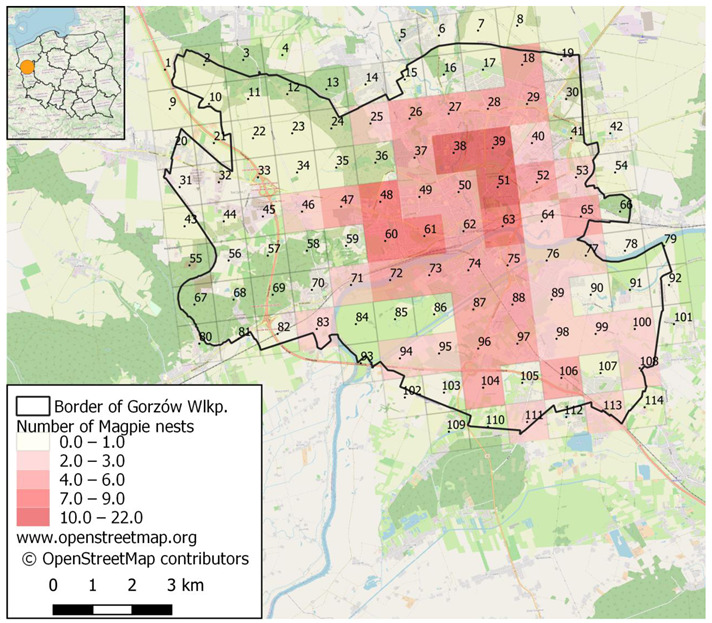
Spatial distribution and density of Magpie nests in the study area, divided into squares (*n* = 114) of 1 × 1 km.

For the study, the city area was divided into 114 squares of 1 × 1 km (Figure 1) with centroids defined by QGIS 3.6 software (QGIS.org 2021). Designated centroids were used due to the lack of geographic coordinates of particular nests in the squares. Taking the data from the Urban Atlas 2012 [16] and using QGIS software for each square, we calculated the area of the following types of habitats: green urban areas, sport and leisure grounds, arable land, pastures, forests, herbaceous vegetation associations, wetlands and water (i.e., natural ones) and areas covered with multi-family buildings, old town, and industrial areas. Land use classes for each habitat type are listed in Appendix A. As a measure of degree of urbanization, for each square, we calculated the percentage contribution of sum of areas of all urbanized habitats. For the habitat gradient analysis, only squares entirely within the city boundaries were used (a total of 58 squares).

### 2.2. Data Collection

The data were collected in two sessions in March and April 2014, aiming to detect the highest number of present pairs. The research time in a particular square ranged from ca. 10 to 90 min and was dependent on several factors: the area of the square was located only within the city limits; availability of trees in the open area (fields and meadows); share of forest areas in the square (such sites were searched, especially in ecotone areas) and compact pine monocultures (except for ecotone zones) were disregarded; single-family housing density and the number of trees available in home gardens; the number of conifers that had to be searched in more detail to assess the existence of occupied nests; activities of adult birds at and around the nest. Experienced researchers paid special attention to the presence of fresh twigs in the nest, the presence of adults with nest material, disturbed birds by the presence of humans at the nest, staying in the nesting area and incubating. The following data were collected: tree species in which a nest was built, tree height and height of the nest placement in the tree, microhabitat type (single tree, tree cluster (2–3 trees, 4–10 trees or more than 10 trees), row of trees and additional descriptions of the microhabitats, i.e., park, allotment gardens, cemeteries, near open water). Observations were carried out with binoculars. The tree height and the nest height placement were determined by a Suunto altimeter PM5/1520 and visual method with accuracy to 0.5 m. For the spatial analysis, qGIS 3.6 software was used.

### 2.3. Statistical Analysis

We calculated Pearson’s correlation coefficient to explore the relationship between Magpie nest density and degree of urbanization in Gorzów Wlkp. In order to explore the environmental characteristics affecting the nest number of *Pica pica* in the city of Gorzów Wlkp., we run generalized linear models (GLM) [17]. We used Pearson’s product–moment correlations to explore the relationships among predictors, avoiding too redundant covariates in the models. Only predictors with Pearson’s product–moment correlations below 0.6 were included, in order to avoid multi-collinearity [18]. Then, we excluded the variable nest height, because it correlated with the mean tree height (R^2^ = 0.98, *p* = < 0.001).

The first model was run accounting for variation in number of nests in each 1 × 1 km square, in relation to distance from the external border of the urban area and mean tree height. The model was fitted assuming a Poisson distribution for number of nests, after determining the type of distribution of variable [19] using the package ‘MASS’ [20]. The second model was run accounting for variation in nest height in relation to distance from the external border of the urban area and mean tree height. The model was fitted assuming a normal distribution for nest height. It was not necessary to transform the response variables in the models. Akaike’s Information Criterion (AIC) was used to determine the model that ‘best’ explained variation in the data [21].

All statistical tests were performed with R software version 3.6.0 [22].

## 3. Results

**Figure 2:**
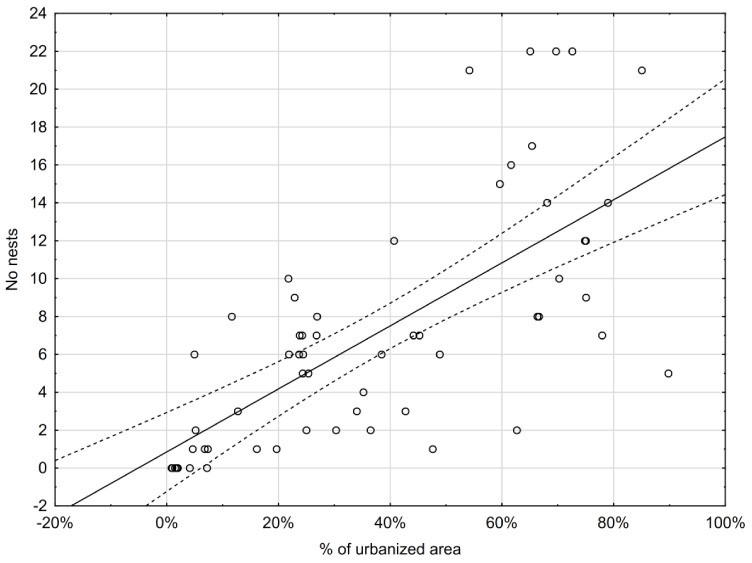
Correlation of Magpie nest density and degree of urbanization in Gorzów Wlkp. (r = 0.700).

A total of 474 Magpie pairs and their occupied nests were recorded in all the 114 squares covering the whole city area (Figure 1., Appendix B). The average density amounted to 5.5 pairs/km^2^ (min = 0, max = 22 nests/square). The density of the Magpie in the squares entirely situated in Gorzów Wlkp. was 6.9 p/km^2^, in the nonurbanized habitat type—3.7 p/km^2^, and in the urbanized habitat type—13.5 p/km^2^. The correlation of nest density with the urbanization gradient was highly significant (r = 0.700, *p* < 0.0001) (Figure 2).

Magpie nests were found in the following tree species and proportion: *Picea* sp. 20.1%; *Populus* sp. 19.5%; *Betula* sp. 12.5%; *Acer* sp. 11.2%; *Salix* sp. 10.2%; *Robinia* sp. 8.7%; Unmarked 5.1%; *Tilia* sp. 3.2%; *Larix* sp. 1.5%; *Prunus* sp. 1.5%; *Alnus* sp. 1.1%; *Quercus* sp. 1.1% and others (Appendix B).

The mean nest height was 11.5 m (SD: 4.8 m, max: 30 m, min: 3 m), whilst the mean trees height used for nesting was 13.4 m (SD: 5.5, max: 33 m, min: 4 m).

**Figure 3:**
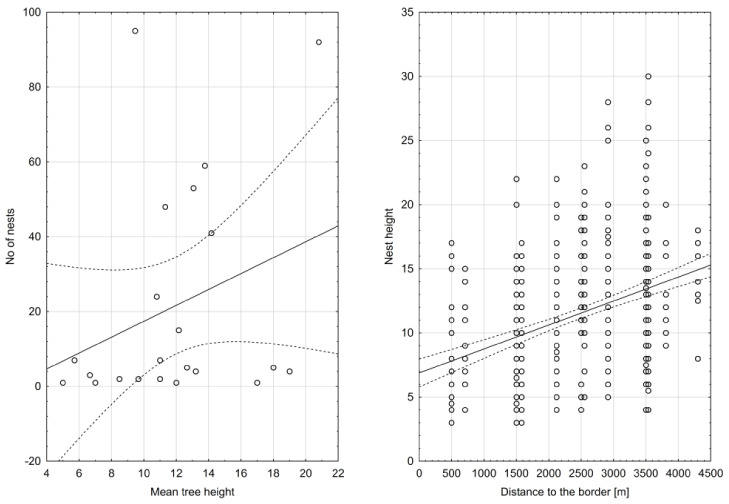
Regression between number of nests and mean tree height. (left) Nest height = −0.315 + 0.878 × mean tree height; R^2^ = 0.97, and number of nests and mean distance to the city border. (right) Nest height = 6.601 + 0.002 × distance to the border; R^2^ = 0.21.

The mean distance from the centroid of nest sites to the external border of the urban area of the city of Gorzów Wlkp. was 1.976 m (SD: 1.021 m, max: 4300 m, min: 500 m). Trees most frequently used for nesting were arranged in rows (26.3%), followed by single trees (24.6%) and clusters of 4–10 trees (20.1%) and this difference was statistically significant (Chi^2^ = 50.2; df = 4, *p* < 0.01). There is a positive significant correlation between the mean nest height placement and mean tree height and distance to the border of the city (Figure 3, Table 1). Additionally, a significant positive correlation between the mean height of the nest placement and the mean tree height was found (Figure 3, Table 1).

## 4. Discussion

Magpie populations are stable or slightly declining in some European countries, increasing with degree of urbanization [6,23]. Magpie abundance increased in all towns studied in western Poland over the past 50 years [6]. Suburban populations grew the fastest, but the highest density occurred in urban populations [6]. Šálek et al. [23] point to the need for research across urbanization gradients, which are evaluating the effect of human activity or intensity of urbanization on breeding biology, especially breeding adaptations in highly anthropopressured areas. Our research results from Gorzów Wlkp. bring valuable insights into this issue.

In the second half of the XXth century, Magpies showed a spectacular colonization of Eurasian towns [15]. Studies on urbanization of bird populations provide important information about the causal factors behind the regulation of bird numbers [15]. The Magpie density in Gorzów Wlkp. found in the present study (5.5 p/km^2^) is lower than in 1989, although the city area has increased [24], and is similar to the average density in other cities in Poland, e.g., in Siedlce—2.1 [4]; Lublin—4.3 [25]; Szczecin—8.7 p/km^2^ (after [6]) and several times lower than in the cities with the highest density in Poland: Zielona Góra—up to 31.1 p/km^2^ [26], Wrocław (1.1 to 46 p/km^2^ depending on the selected zone [11]). Compared to Central European cities, the density is also several times lower (17–57 p/km^2^) [6,27].

The highest Magpie density occurs in the most populated parts of the city, i.e., downtown and in multi-family housing areas, and the lowest in non/less urbanized areas. The increasing percentage of the number of nests along the urbanization gradient shows that the Gorzów Wlkp. Magpie population has undergone the synurbanization process and is currently in the “spread” phase. The ecological costs of exposure to human disturbance, i.e., also to domestic cats, probably are acceptable, taking into account the ecological benefits in terms of living in an urban habitat and access to food and shelter. The flight initiation distance of magpies decreased and birds learned to use anthropogenic factors (especially food availability, artificial materials for nest building) to grow their population. Similar results for Magpie populations along an urbanization gradient were detected in many cities, e.g., Białystok, Olsztyn, Gdańsk, Sofia, Berlin, Rovaniemi, České Budějovice [3,5,11,23,28,29]. Due to the decrease in persecution, the Magpie has habituated to the constant presence of humans and traffic in urban areas [6] and takes advantage of the almost constant food availability, especially in winter time [11]. In the city, Magpies can use abundant breeding sites, which allows successful reproduction and they successfully exploit urban environments, partially due to adaptation of their nesting behaviour [23]. Loss of biodiversity due to urbanization has been well-documented across multiple taxa [30]—especially sensitive to the increase in anthropopressure are pollinators [31]. On the other hand, the more flexible species, such as Corvids, can become indicators for biodiversity-friendly managed urbanized areas and show the direction of adaptation in urbanized measures.

Our results confirm that Magpie are flexible in tree species selection in the breeding period (21 taxa). The number of taxa of nest trees in Gorzów Wlkp. was very similar to that reported from other Polish cities [4,26,32]. Our study shows that the Magpie most often chose spruce *Picea* sp. as a breeding site, followed by poplars *Populus* sp. In contrast, many studies indicate that the vast majority of nests are built on poplars [5,10,15,23,26,33,34,35]. However, an increasing share of conifers has been recorded in recent years by several investigations [5,11,36], which is related to three factors. First, coniferous trees are evergreen and provide good shelter already in early spring, at the beginning of the breeding season. Second, the share of conifers is increasing, because they are willingly planted by the city residents. Third, it has been shown, however, that the Magpies nesting on coniferous species start breeding much earlier [37]. Moreover, the preference for conifers as nest sites may thus be an antipredator behaviour against species such as the Hooded Crow by providing cover in early spring time [11].

The height of the nest site is correlated with the height of the nest tree and also increased with the degree of urbanization. The average height of the nest site in Gorzów Wlkp. was lower than reported for other areas from Poland [2], but comparable with the data from Zielona Góra (average 12.0 m) [26], which could be the result of city structures in western Poland and the availability of lower trees in single-family housing estates in new urbanized areas built and developed in the beginning of the XXI century. This issue requires further research, especially in the historical planning context. The obtained results of the Magpies’ environmental preferences do not differ from the general patterns described earlier [38]. The Magpies’ nest height differed significantly across habitats, and it increased significantly with urbanization intensity and the border of the city. The Magpies did not use urban nests sites randomly, but in a way that maximizes the use of the new habitats available, e.g., conifers. The species is also very flexible in nesting behaviour [15]. However, changes in preference for a specific nest site height do not have to be caused exclusively by minimizing human disturbance. Most likely, the preference for a high degree of urbanization is a compromise of the following factors: rather low pressure of predators in city centres, a low energy expenditure for food and reproduction, and de facto reduction in flight zone and living in an increasingly close human neighbourhood. In the city of Gorzów Wlkp., Magpies built nests mainly in a tree rows (26.3%) and on free standing single trees (24.6%). Similar breeding places are chosen in the extra-urban landscape [26,28,32,35,39].

## 5. Conclusions

The increasing proportion of the number of Magpie nests with increasing urbanization shows that Magpie population in Gorzów Wlkp. has undergone the synurbanization process and is currently in the “spread” phase. The obtained results of the magpies’ environmental preferences do not differ from the general patterns described earlier. The study shows that Magpies can adapt to changing urbanization factors, and the choice of conifers helps the species to adapt to highly anthropogenic habitats.

## Figures and Tables

**Table 1:** Results of GLM accounting for number of nest and mean nest height in each (1 × 1 km) square in the city of Gorzów Wlkp. in relation to the distance from the external border of the urban area and the average tree height. Confidence intervals for number of the nest: LL = lower level. UL = upper level. AIC: 132.28. AIC of the best model: 487.84. Confidence intervals for mean nest height: LL = lower level. UL = upper level. AIC of the best model: 132.28.

Variables	Estimate	Robust SE	Pr(>|z|)	LL	UL
(Intercept)	0.48955	0.220202	0.0262	0.057955	0.921146
Distance to the border	0.000405	<0.001	<0.001	0.00026	0.000549
Tree height	0.049789	0.022938	0.0300	0.004831	0.094747
(Intercept)	−0.35433	0.197455	0.07273	−0.74134	0.03268
Distance.border.m.	<0.001	<0.001	0.44917	<0.001	0.000189
Tree.height.mean.	0.872784	0.018679	<0.001	0.836173	0.909395

## Data Availability

Not applicable.

## Appendix A. The Proportion of Particular Habitat Types in Gorzów Wlkp

**Land Use**	**Urban Atlas Code**	**Area [ha]**	**%**
Green urban areas	14100	394.0	4.6%
Sports and leisure facilities	14200	419.1	4.9%
Arable land (annual crops)	21000	1978.9	23.1%
Pastures	23000	1792.5	20.9%
Forests	31000	679.6	7.9%
Herbaceous vegetation associations	32000	381.3	4.4%
Wetlands	40000	9.3	0.1%
Water	50000	126.4	1.5%
Continuous Urban Fabric	11100	455.5	5.3%
Discontinuous Dense Urban Fabric	11210	687.0	8.0%
Discontinuous Medium Density Urban Fabric	11220	90.0	1.0%
Discontinuous Low Density Urban Fabric	11230	4..2	0.0%
Discontinuous very low density urban fabric	11240	51.0	0.6%
Isolated Structures	11300	51.6	0.6%
Industrial, commercial, public, military and private units	12100	903.6	10.5%
Fast transit roads and associated land	12210	28.4	0.3%
Other roads and associated land	12220	314.9	3.7%
Railways and associated land	12230	63.7	0.7%
Mineral extraction and dump sites	13100	60.6	0.7%
Construction sites	13300	68.7	0.8%
Land without current use	13400	11.7	0.1%
**Total:**		8572.1	100.0%

## Appendix B. Genera or Species of Trees and Shrubs Used by Magpies for Nest Building in Gorzów Wlkp. in 2014

**Tree Species**	**Microhabitat**							**Additional Description of the Environment**				**Mean Tree Height; Maximum;** **Minimum;** **Standard Deviation**	**Mean Nest Height Location; Maximum;** **Minimum; Standard Deviation**
	**Single Tree**	**Tree Cluster 2-3**	**Tree Cluster 4-10**	**Tree Cluster > 10**	**Row of Trees**	**Unclassfied**	**Park**	**Allotment Gardens**	**Cementary**	**Near Open Water**	**Unclassified**		
*Acer* sp. 11.2%	14	10	5	5	13	6	9	3		1	40	13.1; 21.0; 5.0; 4.1	11.9; 4.0; 19.0; 3.6
*Aesculus* sp. 0.8%	2	1				1	1			1	2	13.2; 15.0; 10.0; 2.5	10.7; 9.0; 13.0; 2.1
*Alnus* sp. 1.1%	0		4	1						1	4	12.7; 15.0; 10.0; 2.6	10.0; 7.0; 12.0; 2.3
*Betula* sp. 12.5%	15	12	15		15	2	1	1	2	1	54	13.8; 20.0; 5.0; 3.9	11.4; 4.0; 20.0; 3.5
*Carpinus* sp. 0.8%	1	1			1	1	1				3	19.0; 20.0; 17.0; 1.7	16.8; 14.0; 19.0; 2.2
*Corylus* sp. 0.2%	0		1								1	5.0; 5.0; 5.0; -	4.0; 4.0; 4.0; -
Crataegus sp.0.2%	1										1	12.0; 12.0; 12.0; -	10.0; 10.0; 10.0; -
*Fraxinus* sp. 0.2%	0		1								1	7.0; 7.0; 7.0; -	4.0; 4.0; 4.0; -
*Juglans* sp. 0.4%	1		1								2	11.0; 11.0; 11.0; -	9.0; 9.0; 9.0; -
*Larix* sp. 1.5%	3	2	1		1						7	11.0; 14.0; 8.0; 2.8	9.8; 7.0; 13.0; 2.2
*Malus* sp. 0.6%	1		1			1		1			2	6.7; 8.0; 5.0; 1.5	5.3; 4.0; 6.0; 1.2
*Picea* sp. 20.1%	19	20	39		15	2	1	22	1		71	9.5; 18.0; 5.0; 3.2	8.1; 4.0; 15.0; 2.5
*Pinus* sp. 0.4%	0		2					1			1	9.7; 12.0; 5.0; 4.0	8.3; 4.5; 11.0; 3.5
Platanus sp. 0.2%	0				1						1	17.0; 17.0; 17.0; -	16.0; 16.0; 16.0; -
*Populus* sp. 19.5%	22	17	2	1	47	3	1		3	4	84	20.8; 33.0; 10.0; 5.0	17.3; 8.0; 30.0; 4.2
*Prunus* sp. 1.5%	1		2		1	3		3			4	5.7; 7.0; 4.0; 1.1	4.3; 3.0; 6.0; 1.1
*Pyrus* sp. 0.4%	0		1		1						2	8.5; 13.0; 4.0; 6.4	7.5; 4.0; 11.0; 4.9
*Quercus* sp. 1.1%	2		1		2						5	18.0; 27.0; 11.0; 8.2	14.8; 10.0; 22.0; 5.4
*Robinia* sp. 8.7%	5	9	3	9	9	6	6	2	1		32	14.2; 22.0; 5.0; 4.1	12.5; 4.0; 20.0; 3.8
S*alix* sp. 10.2%	16	9	13	2	8			2		6	40	11.3; 19.0; 4.0; 3.7	9.3; 3.0; 17.0; 3.4
*Tilia* sp. 3.2%	4		1	1	7	2	1		1		13	12.2; 16.0; 8.0; 2.2	10.7; 4.0; 15.0; 2.7
Unmarked 5.1%	9	4	2	4	3	2	2	7		1	14	10.8; 19.0; 5.0; 3.5	9.8; 4.0; 16.0; 3.1
**Total [%]**	**116 [24.6]**	**85 [18.0]**	**95** **[20.1]**	**23 [4.9]**	**124 [26.3]**	**29 [6.1]**	**23 [4.9]**	**42 [8.9]**	**8 [1.7]**	**15 [3.2**]	**384 [81.4]**	**13.4; 33.0; 4.0; 5.5**	**11.5; 30.0; 3.0; 4.8**
